# Combined Bioinformatics Analyses and Immunohistochemical Validation Reveal the Prognostic Relevance and Immune-Related Role of CIITA in Breast Cancer

**DOI:** 10.7150/jca.118933

**Published:** 2025-07-28

**Authors:** Wenge Li, Xin Yu, Yue Xia, Juanjuan Li

**Affiliations:** 1 Department of Breast and Thyroid Surgery, Renmin Hospital of Wuhan University, Wuhan, Hubei, P. R. China.; 2 Department of urology, Renmin Hospital of Wuhan University, Wuhan, Hubei, P. R. China.

**Keywords:** breast cancer, CIITA, bioinformatics, tumor immunity, prognostic biomarker.

## Abstract

Breast cancer remains a major global health burden, necessitating improved prognostic markers and therapeutic strategies. This study investigates the role of class II major histocompatibility complex transactivator (CIITA), a master regulator of major histocompatibility complex class II(MHC-II) gene expression, in breast cancer. Although CIITA is well recognized for its role in antigen presentation in immune cells, its function in tumor immunity and prognosis remains underexplored. Through integrative bioinformatics analyses using The Cancer Genome Atlas (TCGA) and other datasets, we demonstrate that high CIITA expression is associated with favorable clinical outcomes and enhanced immune activation in breast cancer. CIITA levels correlate with increased infiltration of antitumor immune cells, elevated expression of immune checkpoint genes, and enrichment of immune-related pathways. Immunohistochemical staining of breast cancer tissues further confirms CIITA protein expression patterns. Moreover, functional enrichment analyses suggest that CIITA may influence tumor-immune interactions by modulating immune response pathways. A prognostic nomogram incorporating CIITA expression shows robust predictive value for overall survival, offering potential clinical utility. These findings highlight CIITA as a promising prognostic biomarker and immunomodulatory target in breast cancer, shedding light on its role in shaping the tumor immune microenvironment.

## Introduction

Globally, breast cancer persists as the predominant malignancy diagnosed in women and the principal contributor to cancer-associated deaths, with annual incidence surpassing two million new cases 1-3. Despite significant progress in early detection and therapeutic modalities—encompassing surgical intervention, cytotoxic agents, hormonal agents, and immunomodulation—a significant subset of breast cancer patients ultimately develop recurrent or progressive disease. This persistent clinical challenge necessitates the identification of reliable prognostic biomarkers and innovative therapeutic approaches 4-6.

Functioning as the principal orchestrator of major histocompatibility complex class II (MHC II) gene expression, class II major histocompatibility complex transactivator (CIITA) operates as a non-DNA-binding transcriptional coactivator 7,8. CIITA critically enables antigen processing and presentation - central to immune surveillance - through its coordination of transcriptional complex assembly at MHC II promoters. This mechanism proves particularly essential for CD4⁺ T lymphocyte activation 9. Fundamentally required for specialized antigen-presenting cell (APC) functionality—including dendritic cells and macrophages—CIITA expression undergoes stringent control via interferon-γ (IFN-γ) signaling pathways 10. Beyond its established functions in adaptive immunity, CIITA demonstrates significant contributions to anti-tumor immunity. This occurs through enhancing tumor immunogenicity and dynamically coordinating local immune responses within the tumor microenvironment (TME), as evidenced by accumulating research.

Within oncological contexts, CIITA augments tumor cell recognition by the immune system through upregulation of MHC II surface molecules, thereby facilitating efficient antigen presentation to CD4⁺ T lymphocytes 11-13. Beyond fostering CD4⁺ T lymphocyte recruitment and activation, this process critically bolsters CD8⁺ cytotoxic T cell priming and expansion. Such enhancement arises via optimized antigen cross-presentation mechanisms coupled with cytokine-mediated signaling support.

Breast cancer represents a heterogeneous disease with variable immune infiltration across molecular subtypes, and recent studies have highlighted the prognostic and therapeutic significance of immune-related signatures in this malignancy. Nevertheless, the specific role of CIITA in breast cancer remains largely unexplored. In particular, how CIITA expression correlates with immune cell infiltration, MHC II expression levels, and patient outcomes across different breast cancer subtypes has not been systematically addressed. Given its central role in immune regulation, CIITA may serve as a valuable biomarker for stratifying tumors based on immune contexture and predicting responsiveness to immunomodulatory therapies.

Here, we comprehensively investigate the expression landscape of CIITA in breast cancer using publicly available multi-omics datasets, and examine its association with tumor-infiltrating immune cells, CIITA gene expression, and clinical prognosis. Our data uncover an overlooked functional interplay between CIITA expression profiles and breast tumor immune phenotypes. This relationship positions CIITA as both a promising prognostic indicator and a potential therapeutic target for modulating anti-tumor immunity.

## Materials and Methods

### Patients and Specimens

TCGA-BRCA Cohort: Employing transcriptomic profiles and clinopathological data from 1,091 breast cancer cases within The Cancer Genome Atlas (TCGA), this investigation retrieved datasets through the UCSC Xena bioinformatics platform. The TCGAbiolinks R package facilitated systematic acquisition of somatic mutation datasets, which were subsequently transformed to Mutation Annotation Format (MAF) via maftools. This standardized preprocessing enabled comprehensive downstream analytical workflows. We used the survminer R package to determine the critical threshold for gene expression.

IHC Cohort: Prospectively collected between 2016-2019, this cohort comprises formalin-fixed paraffin-embedded tissue specimens from 100 breast carcinoma patients treated at Wuhan University Renmin Hospital. Eligibility required ≥ 5-year minimum follow-up. All participants provided written informed consent approved by Renmin Hospital's Institutional Ethics Committee (IEC). Study endpoints encompassed local recurrence or distant metastasis.

### IHC

IHC staining was conducted using standardized procedures following established protocols 14. Specimens received blocking in 3% bovine serum albumin (BSA) for 30 min, a measure to minimize nonspecific binding. Subsequently, endogenous peroxidase was blocked by incubation in 3% hydrogen peroxide for 25 min at room temperature, with light protection. For the reduction of nonspecific binding, a 30-min blocking step using 3% bovine serum albumin (BSA) was applied to the specimens. Overnight incubation at 4°C employed primary antibody specific for CIITA (ABCAM, ab117598) at a 1:100 dilution. Detection employed horseradish peroxidase (HRP) conjugate incubation (50 min, RT); subsequent diaminobenzidine (DAB) staining revealed antigen-antibody complexes. The process was carefully monitored using a microscope. For nuclear counterstaining, specimens were immersed in hematoxylin for 3 minutes. Staining outcomes were assessed based on the intensity and extent of positive neoplastic cell staining. Protein expression intensity was stratified as: 0 (undetectable), 1 (faint light brown), 2 (moderate brown), and 3 (intense dark brown). Final staining scores represented the product of intensity and percentage values.

### Gene Signature Enrichment Score Calculation

Transcriptomic pathway activity was quantified via gene set variation analysis (GSVA) 15. As described previously 16,17, a cancer-relevant gene signature collection was assembled, incorporating KEGG-derived metabolic pathways (MsigDB) and compound-responsive signatures. These signatures depict immune-targeting pathways and forecast radiotherapy responsiveness. A comprehensive compilation is provided in Table S1. PROGENy quantified tumor pathway activity across specimens.

### Identification of Differentially Expressed Genes (DEGs) and Functional Annotation

DEG screening via Limma applied stringent cutoffs (P < 0.05, FC > 1.5). ClusterProfiler executed functional annotation and enrichment analyses, encompassing Gene Ontology (GO) and KEGG pathways. Additionally, gene set enrichment analysis (GSEA) interrogated significant enrichment of GO, KEGG, and Hallmark gene sets.

### Quantifying immune cell infiltration

Breast tumor immune microenvironments were profiled via CIBERSORT to quantify 22 immune cell subtypes. GSVA-derived enrichment scores quantified transcriptomic activity across cancer immunity cycle stages (Xu et al. gene sets 18), characterizing immune progression in individual samples.

### Chemotherapeutic Response Profiling

The pRRophetic algorithm predicted half-maximal inhibitory concentrations (IC₅₀) values of conventional chemotherapy agents.

### CIITA-Integrated Prognostic Nomogram Construction

Multivariate Cox regression assessed CIITA expression and clinical covariates for prognostic biomarker identification. A CIITA-integrated nomogram was constructed via regplot, incorporating age, stage, PAM50 subtypes, and CIITA expression as covariates. This nomogram computes breast cancer survival probabilities by integrating CIITA expression, age, tumor stage, and PAM50 subtypes.

### Statistical Analysis

Pearson correlation matrices assessed inter-variable relationships; normally distributed continuous variables underwent t-tests for inter-group comparisons. Kruskal-Wallis testing determined inter-group differences. Subset heterogeneity was assessed by log-rank tests, with Kaplan-Meier (KM) methodology generating survival curves. SangerBox and R 4.0.0 implemented all statistical analyses; two-tailed P-values established significance (α < 0.05).

## Results

### CIITA Transcriptional Profiles in Breast Carcinomas

**Figure 1:**
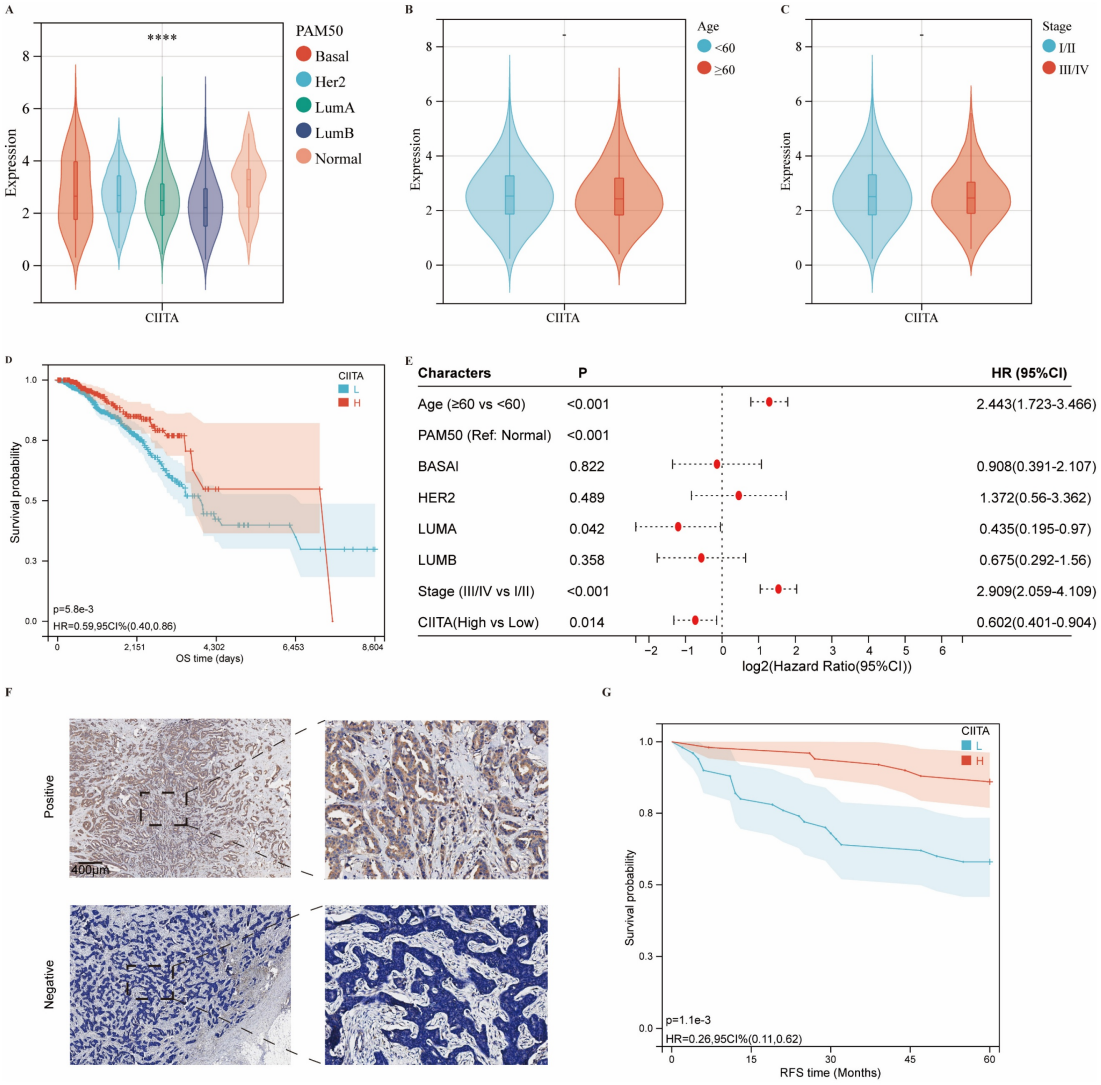
** The expression of CIITA in breast cancer. (A-C)** Transcriptional expression of CIITA stratified by (A) Pam50 subtypes, (B) Age, and (C) Stage. **(D-E)** Kaplan-Meier (D) and Cox regression (E) analyses evaluating the prognostic value of CIITA in overall survival (OS) using TCGA-cohort data. **(F)** Representative immunofluorescence staining images illustrating CIITA expression. **(G)** RFS curves for CIITA in the IHC-cohort. Statistical significance denoted as *p < 0.05, **p < 0.01, ***p < 0.001, ****p < 0.0001.

CIITA expression association with breast cancer clinicopathology was investigated. Differential expression analysis across molecular subtypes revealed significantly elevated CIITA levels in normal-like versus other major subtypes (Basal-like, Luminal A/B, HER2-enriched) (Fig. 1A). However, CIITA levels did not vary significantly with patient age or disease stage (Fig. 1B, 1C). TCGA cohort stratification by CIITA expression dichotomization assessed prognostic relevance. Reduced CIITA expression correlated with inferior outcomes in Kaplan-Meier analysis (Fig. 1D). Multivariate Cox modeling established CIITA as an autonomous breast cancer prognostic determinant (Fig. 1E). Immunohistochemistry in 100-patient specimens confirmed CIITA's preferential epithelial expression with diffuse cytosolic distribution (Fig. 1F). Elevated CIITA expression predicted extended recurrence-free survival (RFS) (Fig. 1G). CIITA demonstrates prognostic biomarker potential in breast cancer.

### DEG Profiling and Functional Enrichment Annotation

**Figure 2:**
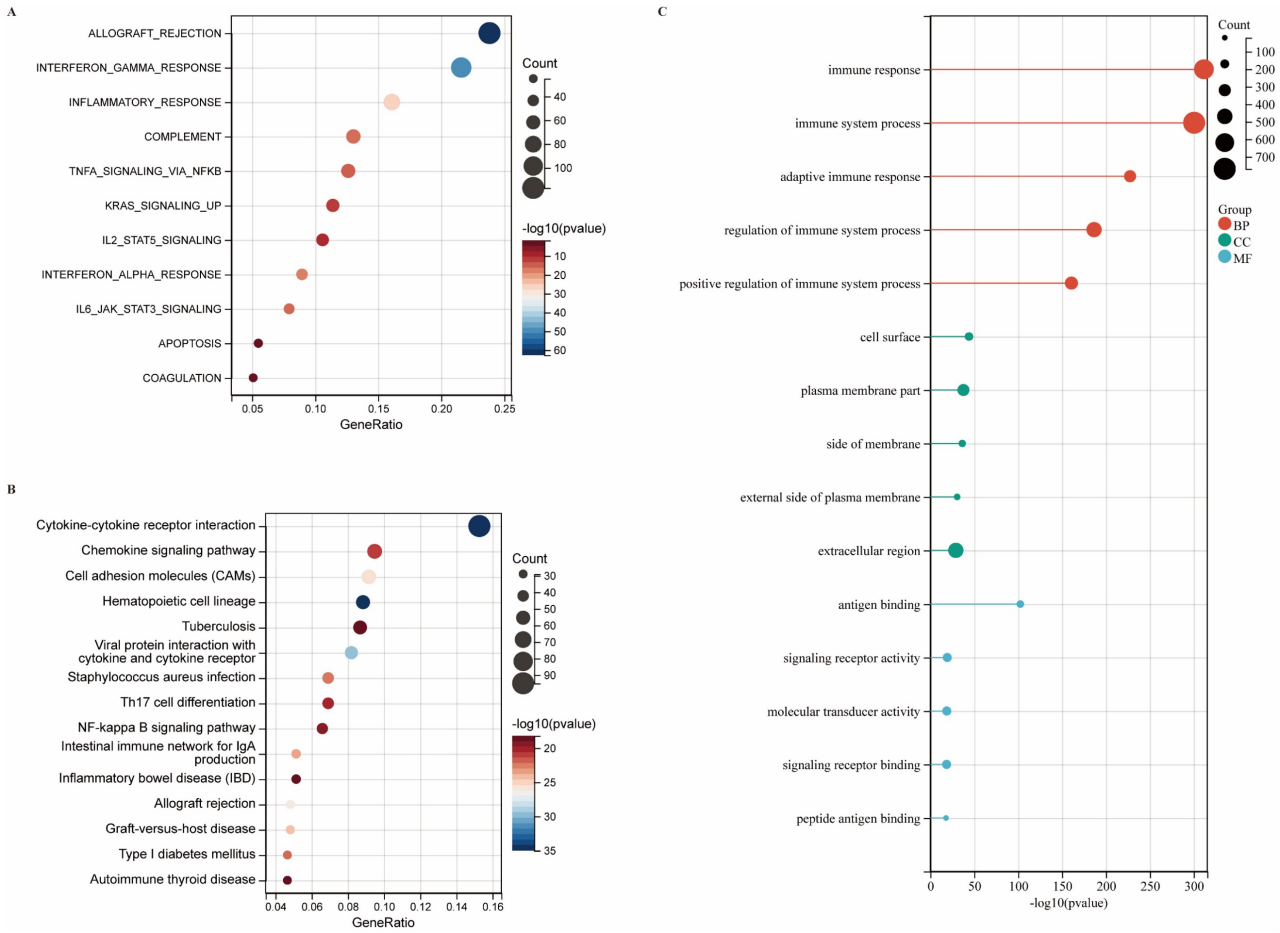
** Identification of DEGs and functional annotations. (A-C)** Enrichment analysis of differentially expressed genes (DEGs) was conducted across various databases: (A) Hallmark gene set analysis, (B) KEGG pathway analysis, (C) GO enrichment analysis.

Transcriptomic profiling identified 1,724 differentially expressed genes (DEGs) in CIITA-high cohorts, comprising 1,592 upregulated and 132 downregulated transcripts (Fig. S1A, B). Biological relevance of DEGs was deciphered through pathway enrichment frameworks. Hallmark profiling linked high CIITA to heightened immune signatures: "transplant rejection," "IFN-γ signaling," and "inflammatory cascades" (Fig. 2A). Upregulated DEGs demonstrated primary enrichment in KEGG pathways: "cytokine-cytokine receptor interaction," "chemokine signaling," and "cell adhesion molecules (CAMs) " (Fig. 2B). GO enrichment revealed significant immune process enrichment: "immune response," "immune system process," "regulation of immune system process," "adaptive immune response" (BP); "plasma membrane part," "extracellular region," "cell surface" (CC); "antigen binding," "signaling receptor activity," "molecular transducer activity" (MF) (Fig. 2C). To further characterize the functional landscape associated with CIITA overexpression, GSEA was performed. The results revealed significant enrichment of KEGG pathways, including “T cell receptor signaling pathway,” “natural killer cell mediated cytotoxicity,” and “cytokine-cytokine receptor interaction” in the high CIITA group (Fig. S2A). Consistent with these findings, Hallmark gene sets such as “allograft rejection,” “IL6-JAK-STAT3 signaling,” and “interferon gamma response” were also markedly enriched (Fig. S2B). In addition, Gene Ontology Biological Process (GO-BP) analysis highlighted enrichment of terms related to adaptive immunity, such as “regulation of B cell activation,” “regulation of B cell proliferation,” and “adaptive immune response” (Fig. S2C). CIITA overexpression defines an immune-activating transcriptome in breast tumors.

### CIITA Expression Dynamics within TIME

**Figure 3:**
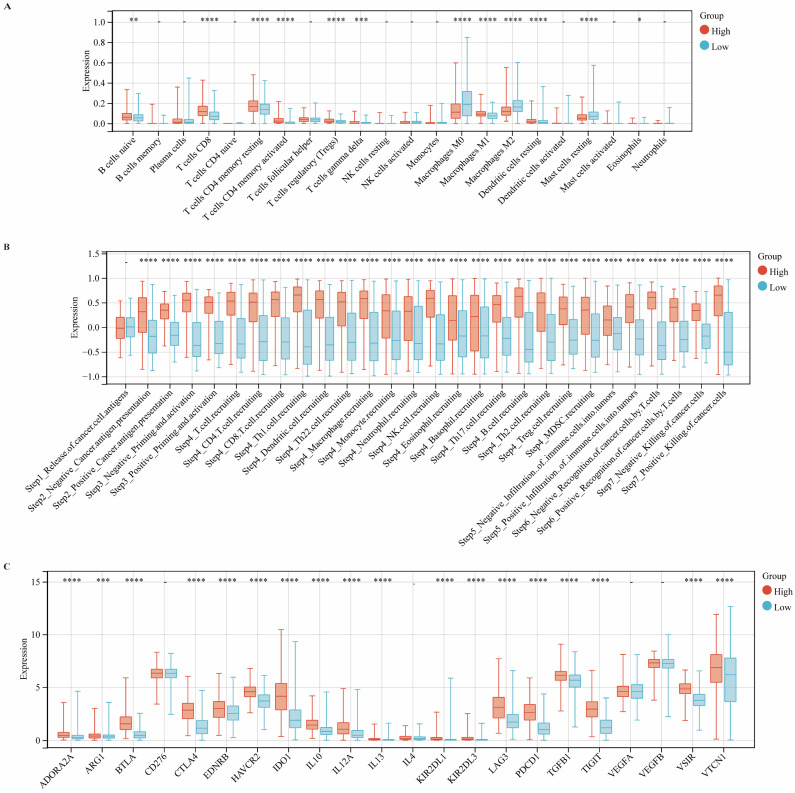
** The expression of CIITA association with TIME. (A-C)** Differential expression of (A) immune cells, (B) immune cycle score, and (C) immune checkpoints in high and low CIITA groups. Significance denoted as *p < 0.05, **p < 0.01, ***p < 0.001, ****p < 0.0001.

CIBERSORT deconvoluted CIITA-associated immune landscapes within the tumor immune microenvironment (TIME) (Fig. 3A). Analysis revealed elevated infiltration of naïve B cells, CD8⁺ T cells, memory CD4⁺ T cells (resting/activated), Tregs, γδ T cells, and M1 macrophages in CIITA-high cohorts; conversely, M0/M2 macrophages and resting mast cells were reduced. Intriguingly, cancer immunity cycle analysis indicated that CIITA scores were positively correlated with both the stimulatory and inhibitory regulatory activities across all stages of the immune cycle (Fig. 3B). Consistently, CIITA expression was also positively associated with the majority of immune checkpoint genes (Fig. 3C). These findings suggest that CIITA plays dual roles in both promoting and restraining anti-tumor immune responses.

### Correlations between CIITA expression and genomic alterations in breast cancer

**Figure 4:**
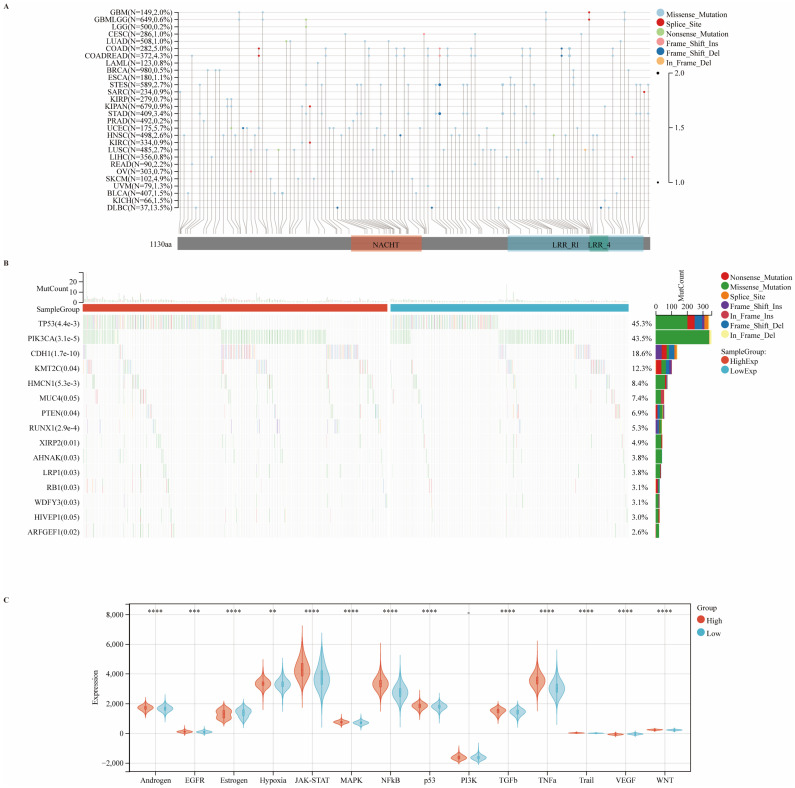
** Carcinogenic Pathways and Mutation Landscape Comparison. (A)** Map position of CIITA mutations in pan-cancer, highlighting mutation frequency and locations within the gene. **(B)** Differential expression of carcinogenic pathways in groups with high and low CIITA. **(C)** Differential expression of carcinogenic pathways in groups with high and low CIITA. Significance indicated as *p < 0.05, **p < 0.01, ***p < 0.001, ****p < 0.0001.

To further elucidate the genomic alterations of CIITA, we systematically assessed its mutation profile across diverse cancer types, with a particular focus on mutation frequency and classification. Our analysis revealed notable heterogeneity in both the frequency and nature of CIITA mutations among various malignancies. Missense mutations emerged as the predominant type across cancer types, with a mutation frequency of 0.5% observed specifically in breast cancer. Although the overall frequency of CIITA mutations remained relatively low, these alterations may nonetheless exert substantial influence on tumor initiation and progression (Fig. 4A). CIITA-high cohorts exhibited elevated TP53/PIK3CA/CDH1 mutation frequencies, predominantly missense variants. These genomic variants may promote aggressive phenotypes via perturbed cell cycle control, signal transduction, and DNA repair mechanisms (Fig. 4B). In the CIITA-high expression cohort, there was a broad activation of several oncogenic signaling cascades, including the p53, WNT, JAK-STAT, and hypoxia pathways (Fig. 4C). Collectively, the data reveal that CIITA expression is linked to specific mutational patterns and the enrichment of pro-tumorigenic signaling pathways, suggesting a broader role for CIITA in influencing the genomic evolution of breast tumors.

### CIITA-Metabolic Pathway Interdependencies

**Figure 5:**
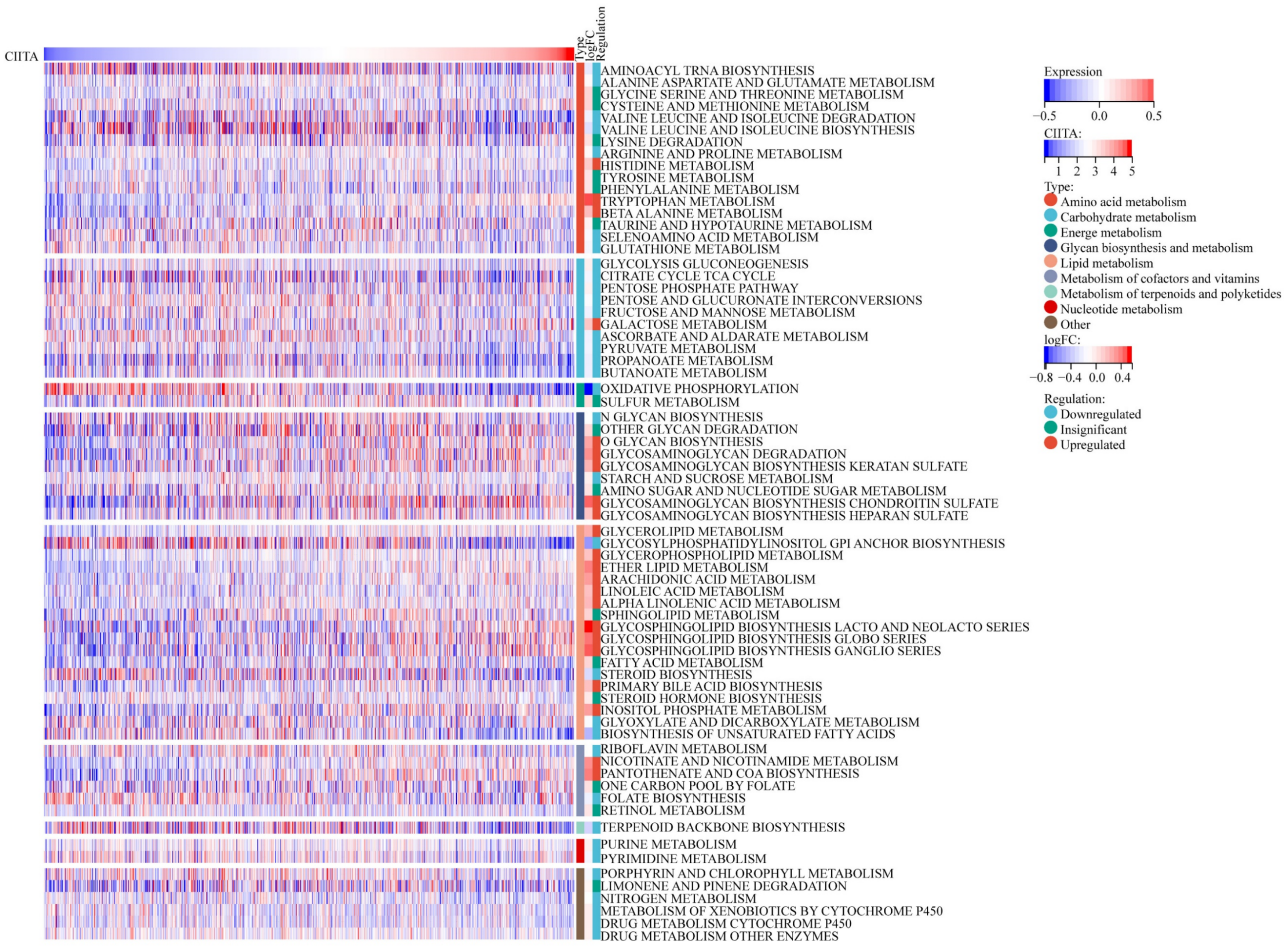
** Correlations between CICTA and metabolism pathways.** The visual representation delineates the expression patterns of metabolic signatures, contrasting the profiles observed in patients with elevated versus reduced CIITA.

Next, CIITA-Metabolic Pathway Interrelationships Were Interrogated (Fig. 5). CIITA-high cohorts exhibited altered metabolic flux across 70 KEGG pathways (22 elevated, 33 suppressed). In patients exhibiting high CIITA expression, several metabolic pathways displayed notable alterations. Specifically, carbohydrate and nucleotide metabolism pathways were markedly suppressed, both of which are recognized as central to malignant tumor progression. Within amino acid metabolism, Next, the association of CIITA with metabolic pathways was explored divergent trends were observed: pathways such as valine, isoleucine degradation, and leucine, as well as arginine and proline metabolism, were significantly downregulated—further underscoring CIITA's association with the inhibition of pro-tumor metabolic programs. In contrast, pathways including arachidonic acid metabolism 19, inositol phosphate metabolism, and nicotinate and nicotinamide metabolism were upregulated. These pathways have been linked to anti-tumor activity and favorable prognosis, particularly when involved in modulating anti-inflammatory responses or constraining metabolic reprogramming in cancer cells. Collectively, the data reveal that CIITA modulates breast cancer metabolism by simultaneously suppressing oncogenic metabolic processes and activating pathways linked to anti-tumor immunity and cellular homeostasis.

### CIITA Predicts Therapeutic Opportunities

**Figure 6:**
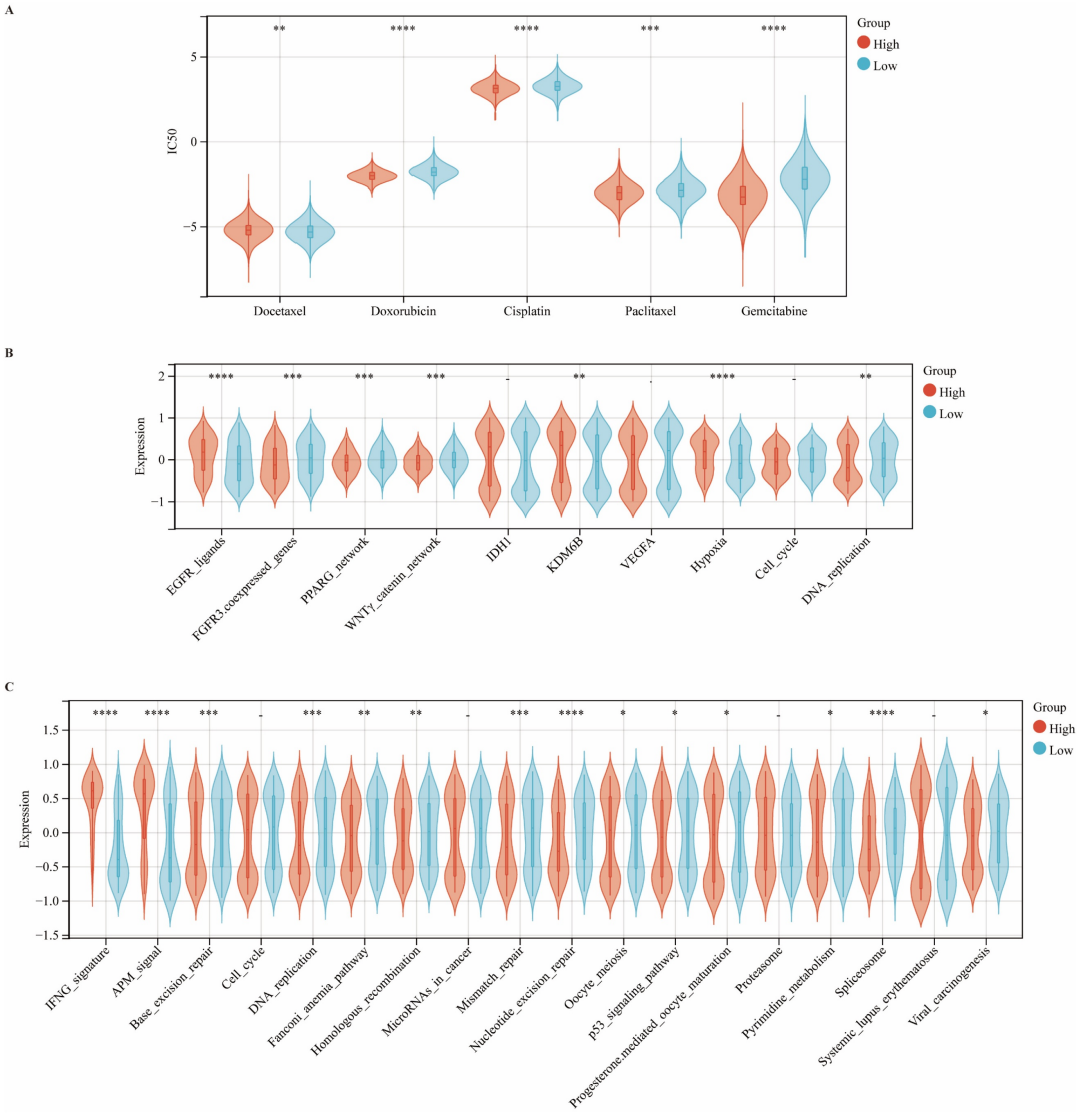
** CIITA predicts therapeutic opportunities. (A)** Association between CIITA expression and chemotherapeutic drug sensitivity. **(B)** Pathway enrichment analysis stratified by CIITA expression. **(C)** Immune and functional pathway modulation by CIITA expression. Significance indicated as *p < 0.05, **p < 0.01, ***p < 0.001, ****p < 0.0001.

We systematically investigated the association between CIITA expression and clinical responses to chemotherapy, radiotherapy, targeted therapy, and immunotherapy. Using the pRRophetic algorithm to estimate drug sensitivity (IC50 values), we observed that patients with high CIITA expression exhibited increased sensitivity to docetaxel, doxorubicin, and cisplatin, but reduced sensitivity to gemcitabine and paclitaxel (Fig. 6A). Moreover, enrichment analysis revealed elevated activity of EGFR ligand signaling and radiotherapy-predictive pathways, including hypoxia and DNA replication, in the high CIITA group. In contrast, the low CIITA expression subgroup exhibited higher enrichment of targetable pathways such as FGFR3-coexpressed genes, the PPARG network, and the WNTγ-catenin network, suggesting that therapeutic modulation of these pathways may enhance treatment efficacy in CIITA-low tumors (Fig. 6B). Similarly, CIITA markedly enhanced the activity of the IFNG signature and antigen processing machinery (APM) signaling pathways, while concurrently suppressing several immune-associated processes, including base excision repair, spliceosome function, and DNA replication (Fig. 6C). Taken together, these data suggest that CIITA expression stratifies breast cancer patients into distinct therapeutic response groups and may inform personalized treatment strategies.

### Development and Validation of a CIITA-Integrated Prognostic Nomogram

**Figure 7:**
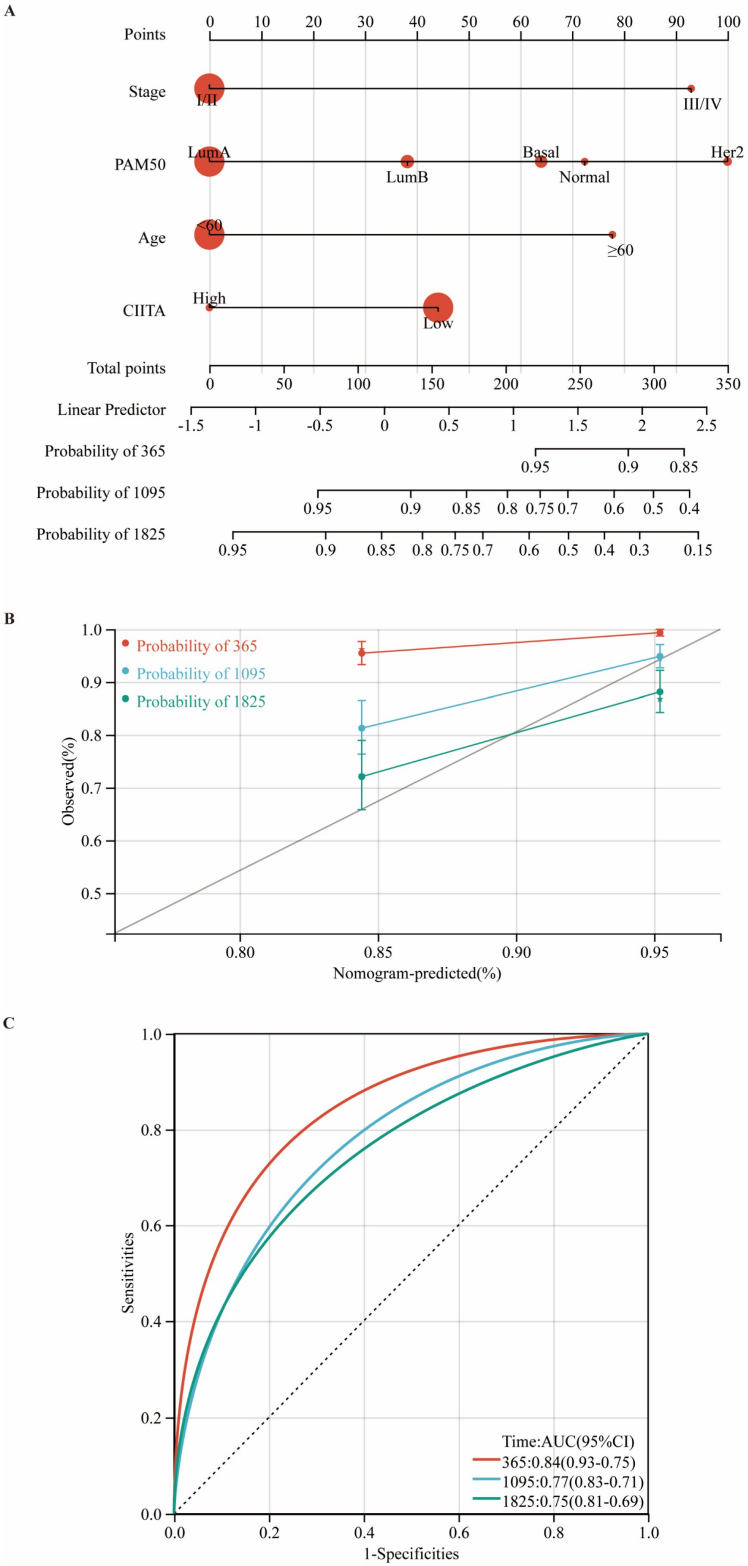
** Construction and validation of a CIITA-based prognostic model. (A)** Prognostic nomogram for overall survival prediction. **(B)** Nomogram predicting overall survival probability using age, Pam50 subtypes, tumor stage, and CIITA expression. **(C)** Calibration curves validating prediction accuracy for 1-year (red), 3-year (blue), and 5-year (green) survival predictions.

Utilizing multivariate Cox regression analysis, we developed a prognostic nomogram for estimating overall survival by integrating key clinicopathological variables: tumor stage, PAM50 subtype, age at diagnosis, and CIITA expression status (Fig. 7A). Calibration plots (Fig. 7B) and time-ROC analyses (Fig. 7C) validated the prognostic tool's precision in clinical outcome prediction.

## Discussion

CIITA was systematically profiled in breast malignancies, delineating its expression dynamics, prognostic significance, and immunological roles. CIITA was identified as a favourable prognostic marker, positively associated with overall survival and enriched in tumors exhibiting heightened antitumor immune responses. These findings underscore the potential of CIITA as an immune-modulatory factor within the breast tumor microenvironment.

These results reinforce prior evidence of CIITA's immunogenic roles in solid malignancies 7,8,20. A study demonstrated that CIITA serves as a master regulator of MHC class II expression and is critical for the induction of tumor immunogenicity through enhanced CD4⁺ T cell activation 4. CIITA expression positively correlated with immune infiltration, notably activated CD8⁺/CD4⁺ T cells and antigen-presenting cells (APCs), indicating proinflammatory microenvironment modulation. These observations align with emerging evidence suggesting that CIITA-induced MHC-II expression on tumor cells facilitates the recruitment and priming of T cells within the tumor microenvironment, thereby augmenting immune surveillance and cytotoxic responses.

Furthermore, our bioinformatic deconvolution of the tumor immune contexture revealed that CIITA expression is associated with immune checkpoints and interferon-γ signalling, hallmarks of an active tumor-immune interface. CIITA-high breast tumors exhibited enhanced expression of antigen processing/presentation, co-stimulatory molecules, and immune effector genes, suggesting an expanded immunoregulatory role beyond MHC-II control. Studies have shown that CIITA's originality and uniqueness lie in its dual functions, not only as a regulator of adaptive immunity, but also as a restriction factor of human retrovirus, which can inhibit virus replication in macrophages 21,22.

Importantly, the therapeutic potential of CIITA modulation extends beyond its role as a prognostic marker. Ectopic CIITA/MHC-II expression reprograms tumor microenvironments by displacing immunosuppressive macrophages and neutrophils with activated CD4⁺/CD8⁺ T cells, bolstering antitumor immunity 23. Hepatocellular carcinoma studies implemented this strategy, engineering multi-epitope/multi-target/multi-allele vaccines to activate CD4⁺/CD8⁺ T cells 24. CIITA induces MHC class II expression in glioblastoma cells, endowing them with surrogate antigen-presenting capability that elicits robust CD4⁺ T cell-mediated adaptive anti-tumor immunity 25.

In addition, the association between CIITA gene mutations and carcinogenesis underscores the importance of maintaining proper CIITA function in the tumor microenvironment 26,27. CIITA mutations may disrupt its regulatory role in immune responses, leading to immune evasion and tumor progression. Triple-negative breast cancer (TNBC) studies by Forero et al. corroborate these findings: elevated CIITA expression correlates with improved progression-free survival (PFS) and enhanced lymphocyte infiltration 4. This suggests that CIITA could serve as a therapeutic sensitizer, particularly in immunologically 'cold' tumors with low CIITA expression.

Our key finding in breast cancer research—that CIITA expression exhibits a significant association with features of the anti-tumor immune microenvironment and patient prognosis—aligns closely with the broader immunomodulatory role of CIITA observed in recent years across diverse malignancies, including lymphoma, melanoma, and colorectal cancer 27-29. This cross-cancer commonality strongly suggests that CIITA is not merely a context-specific regulator, but rather serves as a core modulator of immune homeostasis. Its fundamental mechanism, influencing T cell-mediated anti-tumor immunity through the regulation of antigen presentation machinery such as MHC class II molecules, appears to be conserved across multiple cancer types. Therefore, our study further substantiates the significant scientific value and translational potential of CIITA as a universal biomarker for immunotherapy or a promising therapeutic target.

Several limitations should be acknowledged. This study primarily relies on TCGA data and a relatively small single-center cohort, which may limit generalizability due to sample heterogeneity and regional bias. Although bioinformatics and IHC support the prognostic value of CIITA, functional assays are needed to elucidate its direct role in tumor and stromal biology. The regulatory mechanisms of CIITA and its therapeutic modulation remain uncharacterized. Additionally, limited longitudinal follow-up hinders robust prognostic stratification.

In summary, our integrative analysis identifies CIITA as a pivotal immune regulator in breast cancer, linking its expression to improved clinical outcomes and robust antitumor immunity. Future studies are warranted to explore the therapeutic utility of CIITA modulation in breast cancers and its potential synergy with immune checkpoint blockade or cancer vaccines. The development of CIITA-targeted therapies could revolutionize the treatment landscape for breast cancer, particularly in subtypes with poor immunogenicity and limited response to current immunotherapies.

## Supplementary Material

Supplementary figures.

Supplementary table.

## Abbreviations

APM: antigen processing machinery
BP: biological process
CAMs: Cell adhesion molecules
CC: cell surface
CIITA: class II major histocompatibility complex transactivator
DAB: diaminobenzidine
DEGs: Differentially Expressed Genes
FC: fold change
GO: Gene Ontology
GOBP: Gene Ontology Biological Processes
GSEA: gene set enrichment analysis
GSVA: gene set variation analysis
HRP: horseradish peroxidase
IC50: half-maximal inhibitory concentration
IHC: immunohistochemical
KEGG: Kyoto Encyclopedia of Genes and Genomes
KM: Kaplan-Meier
MAF: Mutation Annotation Format
MF: molecular function
MHC-II: major histocompatibility complex class II
PAM50: Prediction Analysis of Microarray 50
PFS: progression-free survival
ROC: receiver operating characteristic
TCGA: The Cancer Genome Atlas
TIME: tumor immune microenvironment
TME: tumor microenvironment
Tregs: regulatory T cells
