# Supplementary material for: Combined Bioinformatics Analyses and Immunohistochemical Validation Reveal the Prognostic Relevance and Immune-Related Role of CIITA in Breast Cancer
Source: J Cancer. 2025 Jul 28;16(11):3513–24. doi: 10.7150/jca.118933 (PMC12374943; doi:10.7150/jca.118933)
Supplement: Supplementary file 1 — Supplementary figures. [file jcav16p3513s1.pdf]

Figure S1: Identification of DEGs and functional annotations

(A)Among the differential genes, 1592 genes were up-regulated and 132 genes were down-regulated.
(B)Differential gene heat map.

Figure S2: GSEA analysis of the functional map of CIITA overexpression

(A-C) Functional profiling of CIITA was performed using Gene Set Enrichment Analysis (GSEA) with gene sets derived from (A) KEGG pathways, (B) Hallmark gene sets and (C) GO-BP pathways.

A

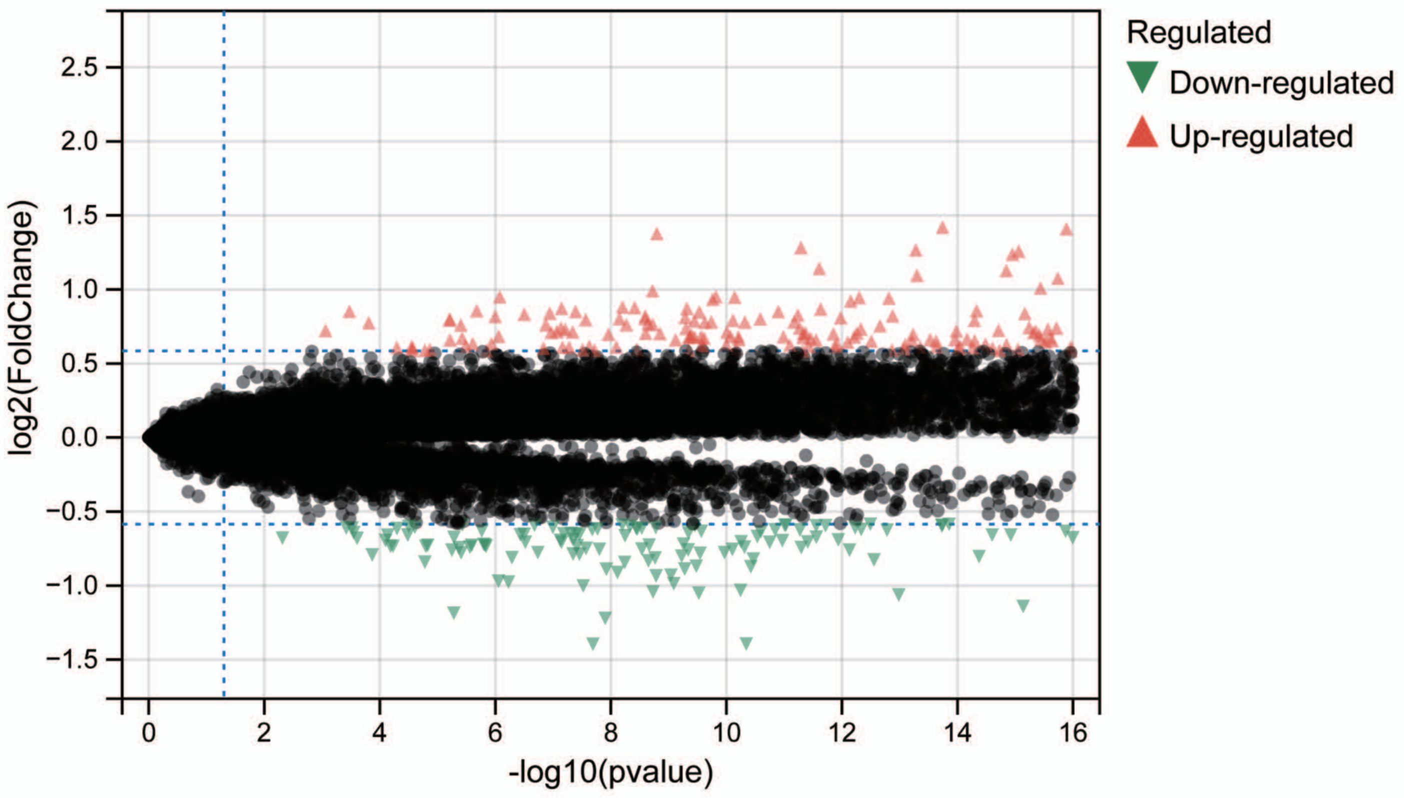
Regulated
Down-regulated
Up-regulated
log2(FoldChange)
-log10(pvalue)
2.5
2.0
1.5
1.0
0.5
0.0
−0.5
−1.0
−1.5
0
2
4
6
8
10
12
14
16

B

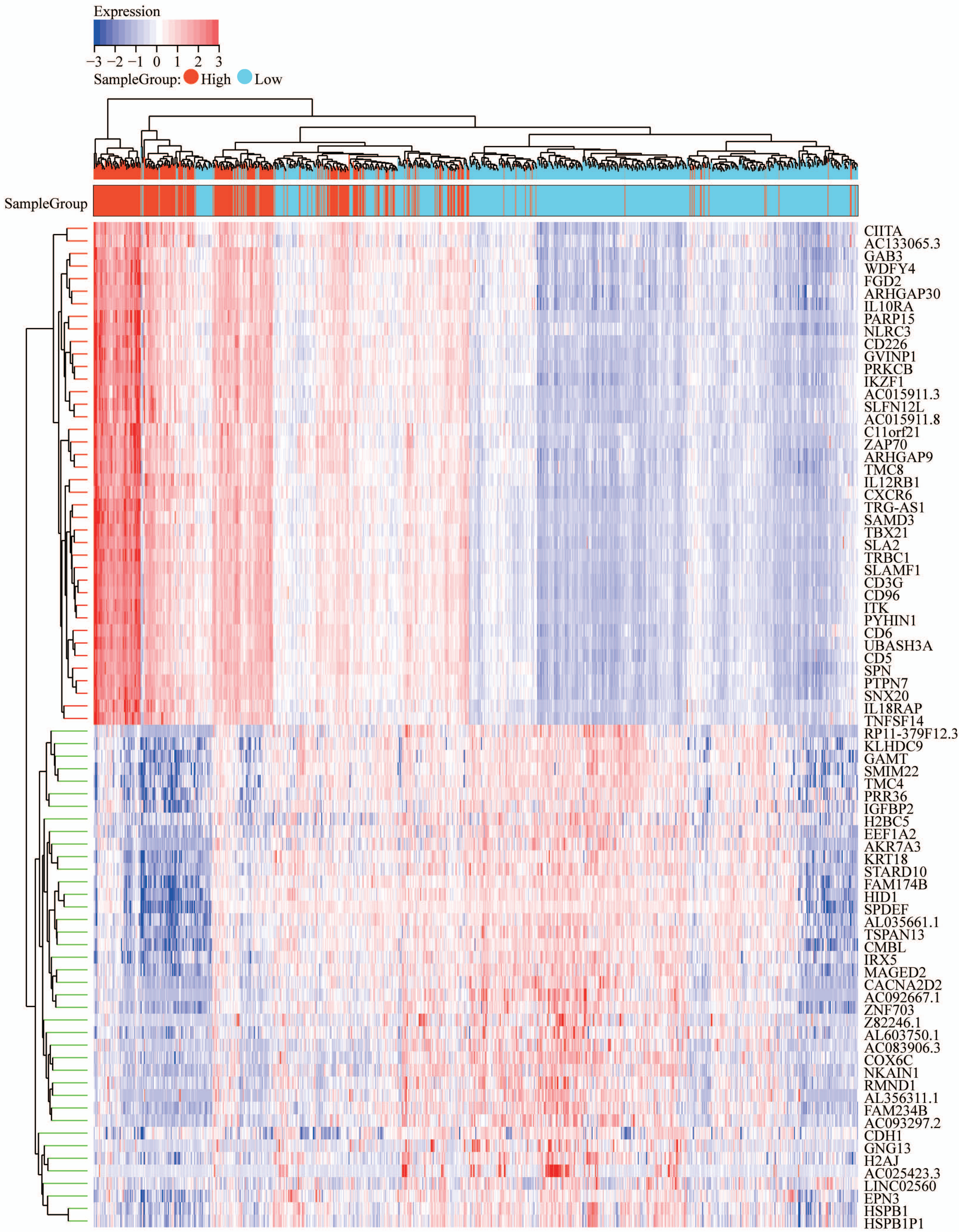
Expression
−3 −2 −1 0 1 2 3
SampleGroup: High Low
SampleGroup
CIITA
AC133065.3
GAB3
WDFY4
FGD2
ARHGAP30
IL10RA
PARP15
NLRC3
CD226
GVINP1
PRKCB
IKZF1
AC015911.3
SLFN12L
AC015911.8
C11orf21
ZAP70
ARHGAP9
TMC8
IL12RB1
CXCR6
TRG-AS1
SAMD3
TBX21
SLA2
TRBC1
SLAMF1
CD3G
CD96
ITK
PYHIN1
CD6
UBASH3A
CD5
SPN
PTPN7
SNX20
IL18RAP
TNFSF14
RP11-379F12.3
KLHDC9
GAMT
SMIM22
TMC4
PRR36
IGFBP2
H2BC5
EEF1A2
AKR7A3
KRT18
STARD10
FAM174B
HID1
SPDEF
AL035661.1
TSPAN13
CMBL
IRX5
MAGED2
CACNA2D2
AC092667.1
ZNF703
Z82246.1
AL603750.1
AC083906.3
COX6C
NKAIN1
RMND1
AL356311.1
FAM234B
AC093297.2
CDH1
GNG13
H2AJ
AC025423.3
LINC02560
EPN3
HSPB1
HSPB1P1

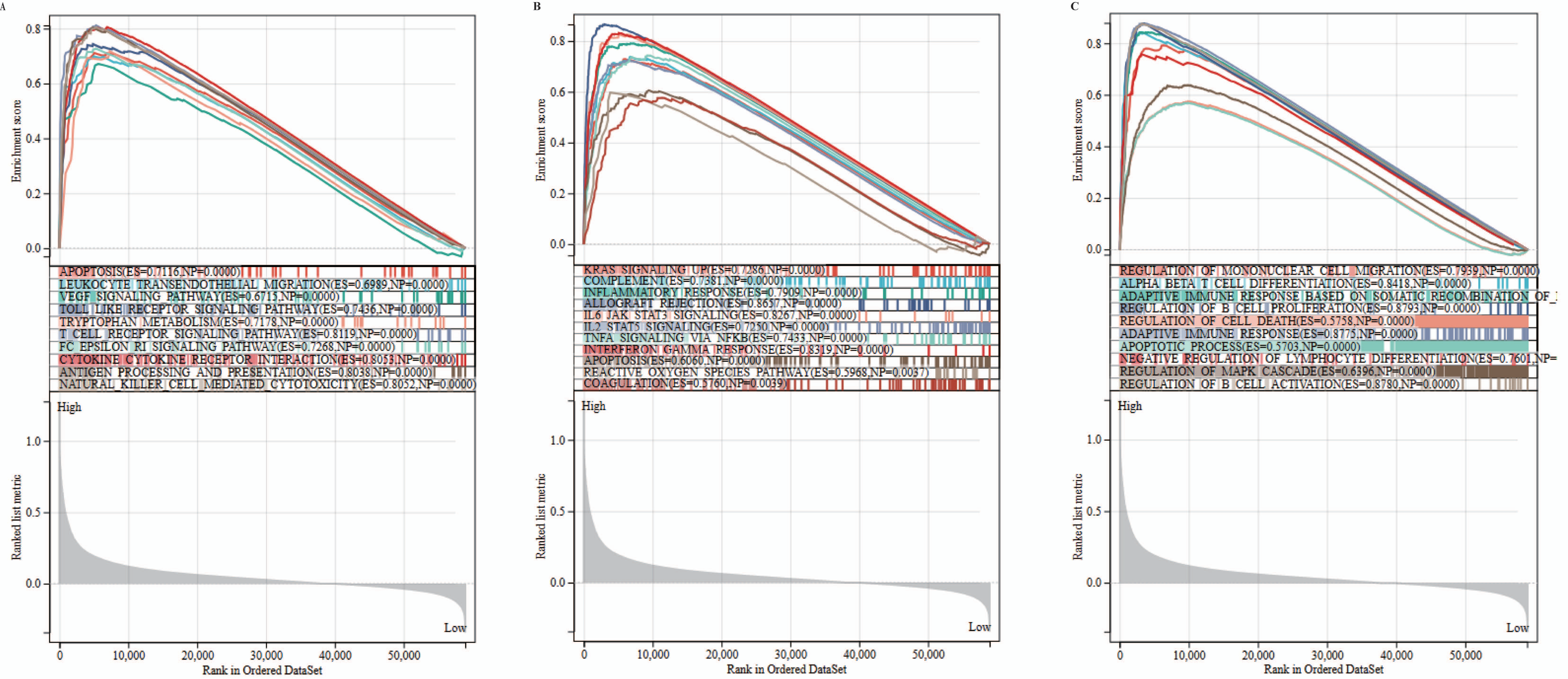

A
Enrichment score
APOPTOSIS(ES=0.7116,NP=0.0000)
LEUKOCYTE_TRANSENDOTHELIAL_MIGRATION(ES=0.6989,NP=0.0000)
VEGF_SIGNALING_PATHWAY(ES=0.6715,NP=0.0000)
TOLL_LIKE_RECEPTOR_SIGNALING_PATHWAY(ES=0.7436,NP=0.0000)
TRYPTOPHAN_METABOLISM(ES=0.7178,NP=0.0000)
T_CELL_RECEPTOR_SIGNALING_PATHWAY(ES=0.8119,NP=0.0000)
FC_EPSILON_RI_SIGNALING_PATHWAY(ES=0.7268,NP=0.0000)
CYTOKINE_CYTOKINE_RECEPTOR_INTERACTION(ES=0.8053,NP=0.0000)
ANTIGEN_PROCESSING_AND_PRESENTATION(ES=0.8038,NP=0.0000)
NATURAL_KILLER_CELL_MEDIATED_CYTOTOXICITY(ES=0.8052,NP=0.0000)
High
Low
Ranked list metric
Rank in Ordered DataSet
B
Enrichment score
KRAS_SIGNALING_UP(ES=0.7286,NP=0.0000)
COMPLEMENT(ES=0.7381,NP=0.0000)
INFLAMMATORY_RESPONSE(ES=0.7909,NP=0.0000)
ALLOGRAFT_REJECTION(ES=0.8657,NP=0.0000)
IL6_JAK_STAT3_SIGNALING(ES=0.8267,NP=0.0000)
IL2_STAT5_SIGNALING(ES=0.7250,NP=0.0000)
TNFA_SIGNALING_VIA_NFKB(ES=0.7433,NP=0.0000)
INTERFERON_GAMMA_RESPONSE(ES=0.8319,NP=0.0000)
APOPTOSIS(ES=0.6060,NP=0.0000)
REACTIVE_OXYGEN_SPECIES_PATHWAY(ES=0.5968,NP=0.0037)
COAGULATION(ES=0.5760,NP=0.0039)
High
Low
Ranked list metric
Rank in Ordered DataSet
C
Enrichment score
REGULATION_OF_MONONUCLEAR_CELL_MIGRATION(ES=0.7939,NP=0.0000)
ALPHA_BETA_T_CELL_DIFFERENTIATION(ES=0.8418,NP=0.0000)
ADAPTIVE_IMMUNE_RESPONSE_BASED_ON_SOMATIC_RECOMBINATION_OF_
REGULATION_OF_B_CELL_PROLIFERATION(ES=0.8793,NP=0.0000)
REGULATION_OF_CELL_DEATH(ES=0.5758,NP=0.0000)
ADAPTIVE_IMMUNE_RESPONSE(ES=0.8775,NP=0.0000)
APOPTOTIC_PROCESS(ES=0.5703,NP=0.0000)
NEGATIVE_REGULATION_OF_LYMPHOCYTE_DIFFERENTIATION(ES=0.7601,NP=
REGULATION_OF_MAPK_CASCADE(ES=0.6396,NP=0.0000)
REGULATION_OF_B_CELL_ACTIVATION(ES=0.8780,NP=0.0000)
High
Low
Ranked list metric
Rank in Ordered DataSet
